# Life‐history traits of the Whiting polyploid line of the parasitoid *Nasonia vitripennis*

**DOI:** 10.1111/eea.12808

**Published:** 2019-07-17

**Authors:** Kelley Leung, Louis van de Zande, Leo W. Beukeboom

**Affiliations:** ^1^ Groningen Institute for Evolutionary Life Sciences University of Groningen PO Box 11103 9700 CC Groningen The Netherlands

**Keywords:** diploid male, triploid female, fitness, body size, parasitization rate, mate competition, biocontrol, Hymenoptera, Pteromalidae

## Abstract

In hymenopterans, males are normally haploid (1n) and females diploid (2n), but individuals with divergent ploidy levels are frequently found. In species with ‘complementary sex determination’ (CSD), increasing numbers of diploid males that are often infertile or unviable arise from inbreeding, presenting a major impediment to biocontrol breeding. Non‐CSD species, which are common in some parasitoid wasp taxa, do not produce polyploids through inbreeding. Nevertheless, polyploidy also occurs in non‐CSD Hymenoptera. As a first survey on the impacts of inbreeding and polyploidy of non‐CSD species, we investigate life‐history traits of a long‐term laboratory line of the parasitoid *Nasonia vitripennis* (Walker) (Hymenoptera: Pteromalidae) (‘Whiting polyploid line’) in which polyploids of both sexes (diploid males, triploid females) are viable and fertile. Diploid males produce diploid sperm and virgin triploid females produce haploid and diploid eggs. We found that diploid males did not differ from haploid males with respect to body size, progeny size, mate competition, or lifespan. When diploid males were mated to many females (without accounting for mating order), the females produced a relatively high proportion of male offspring, possibly indicating that these males produce less sperm and/or have reduced sperm functionality. In triploid females, parasitization rate and fecundity were reduced and body size was slightly increased, but there was no effect on lifespan. After one generation of outbreeding, lifespan as well as parasitization rate were increased, and a body size difference was no longer apparent. This suggests that outbreeding has an effect on traits observed in an inbred polyploidy background. Overall, these results indicate some phenotypic detriments of non‐CSD polyploids that must be taken into account in breeding.

## Introduction

Polyploidy is the heritable condition of having more than the typical number of chromosome sets. It is rare in the animal kingdom but relatively frequent in insects. Polyploid individuals have, for example, been reported from the insect orders Coleoptera, Diptera, Embioptera, Hemiptera, Hymenoptera, Lepidoptera, and Orthoptera (Otto & Whitton, 2000). In particular, Hymenoptera are prone to polyploidy, often as a consequence of a type of sex determination that is common in the order, the ‘complimentary sex determination’ (CSD) mechanism (Cook, 1993; van Wilgenburg et al., 2006; Heimpel & de Boer, 2008). All hymenopterans have haplodiploid sex determination. Males develop from unfertilized eggs and have genomes of maternal origin only, whereas females develop from fertilized eggs and inherit complete chromosome sets from both parents. Accordingly, males are typically haploid (1n) and females are diploid (2n). However, under CSD, increasing numbers of diploid males can arise from inbreeding as a result of homozygosity at *csd* loci. Such males are often sterile, unviable, or sire sterile triploid daughters (Stouthamer et al., 1992; van Wilgenburg et al., 2006) which poses a burden on population growth (Zayed & Packer, 2005) and hampers rearing programs for biological control (Stouthamer et al., 1992).

Polyploids, in particular diploid males, have been recorded for more than 80 species across the hymenopteran tree (van Wilgenburg et al., 2006; Heimpel & de Boer, 2008; Harpur et al., 2013). In many of these cases, the occurrence of polyploidy is linked to the CSD mechanism of sex determination. CSD occurs throughout Hymenoptera, but is clearly absent in some major groups (Stouthamer et al., 1992; van Wilgenburg et al., 2006; Heimpel & de Boer, 2008; Asplen et al., 2009; Elias et al., 2009). Individuals with CSD have either a single *csd* locus or multiple *csd* loci. Those that are heterozygous for at least one *csd* locus develop into females, and those that are homozygous or hemizygous for all *csd* loci develop into males. Inbreeding causes loss of *csd* allelic diversity, increases sterile homozygous diploid male production, reduces population size, which in turn further increases homozygosity. This results in a so called ‘diploid male vortex’ and eventual extinction (Zayed & Packer, 2005; Hein et al., 2009; Zaviezo et al., 2018). Exacerbating this problem is the inability of mating females to discriminate against sterile diploid males (Harpur et al., 2013). Thus CSD species may be more difficult to breed for biological control because of this specific genetic basis of sex determination (Stouthamer et al., 1992).

Less is known about how polyploidy impacts non‐CSD species. Inbreeding does not cause polyploidy in non‐CSD species, and polyploidy in non‐CSD species may not lead to sterile diploid males (e.g., Whiting, 1960; Ma et al., 2015). The absence of CSD is not well documented among the Hymenoptera, but it is especially prominent in the Chalcidoidea, Cynipoidea, and Bethlyoidea parasitoid wasps (Cook, 1993; Cook & Crozier, 1995; van Wilgenburg et al., 2006). As parasitoid wasps are among the most widely used biocontrol agents (van Lenteren et al., 1997; van Lenteren, 2012), knowledge of the potential effects of inbreeding and polyploidy in these taxa is important. Interestingly, polyploidy could potentially also be advantageous for biological control, that is, if polyploidy would confer some fitness advantages, as for example larger yield or hardiness of polyploids in plant breeding (Comai, 2005) and aquaculture (Piferrer et al., 2009). To the authors' knowledge polyploidy has never been explored for beneficial effects on insect breeding.

A case of non‐sterile polyploidy exists in the parasitoid wasp *Nasonia vitripennis* (Walker) (Hymenoptera: Pteromalidae), the most widely studied non‐CSD parasitoid wasp (Beukeboom & Desplan, 2003; Shuker et al., 2003; Beukeboom & Kamping, 2006; Verhulst, 2010; Werren et al., 2010; Verhulst et al., 2013). In rare instances polyploidy has appeared spontaneously in *N. vitripennis* laboratory stocks, although it has not been observed in wild populations or collections (Whiting, 1960; Beukeboom & Kamping, 2006). A ‘Whiting polyploid line’ (WPL) has been maintained in the laboratory since the 1940s, and used primarily for sex determination research (Whiting, 1960; Dobson & Tanouye, 1998; Beukeboom & Kamping, 2006; Beukeboom & van de Zande, 2010; Verhulst, 2010; Verhulst et al., 2013). Diploid males are fertile, produce diploid sperm and sire triploid females. Triploid females have lowered fecundity owing to a high frequency of aneuploid eggs, but they also produce enough viable euploid (haploid and diploid) eggs to continue breeding (Whiting, 1960; Beukeboom & Kamping, 2006).

Despite its long history of research use, much of the Whiting polyploidy strain's baseline biology is unknown. It is the first known available resource for studying viable non‐CSD polyploidy, and highly useful for investigating the potential practical advantages and disadvantages of polyploidy in non‐CSD hymenopterans. Both non‐polyploid and polyploid individuals can be reliably generated for both sexes, and all combinations are viable and capable of reproduction. Furthermore, the breeding scheme of the WPL suggests how non‐CSD polyploids may be used in a biocontrol breeding program. That is, if the female polyploid state confers something beneficial to biological control, this can be reliably passed on through diploid males and a large number of polyploid females can be produced as active biological agents every other generation. Like most commercial hymenopteran lines, the WPL has also become highly inbred over time, so differences between non‐polyploids and polyploids within this strain can be attributed to ploidy state alone, without genetic variation being a contributing factor. These attributes make the WPL a suitable model for a first survey of how polyploidy effects non‐CSD parasitoid wasp breeding.

In this study, we compare life‐history traits of polyploids and non‐polyploids of both sexes from the *N. vitripennis* WPL. Specifically, we compare female and male lifespan under starvation and feeding conditions, male mate competition ability, progeny size and sex ratio, and female parasitization ability. We do this for individuals of both sexes from the long‐used inbred maintenance scheme, as well as for females after a single generation of outbreeding. The latter is a first attempt to discriminate between inbreeding vs. polyploidy effects. In previous studies on other parasitoid wasp species, polyploids were disadvantaged for some traits such as sterility (Stouthamer et al., 1992; van Wilgenburg et al., 2006; Elias et al., 2009) but not for others such as lifespan (Clark & Rubin, 1961) and male mate competition (Elias et al., 2009; Harpur et al., 2013; Thiel et al., 2014), although these studies were exclusively on CSD species. We therefore anticipated that the WPL polyploids might underperform non‐polyploids in some traits, although we could not predict which. We discuss the results in the context of the significance of polyploids in breeding and their performance as biocontrol agents.

## Materials and methods

### *Nasonia* culture and crosses

**Figure 1:**
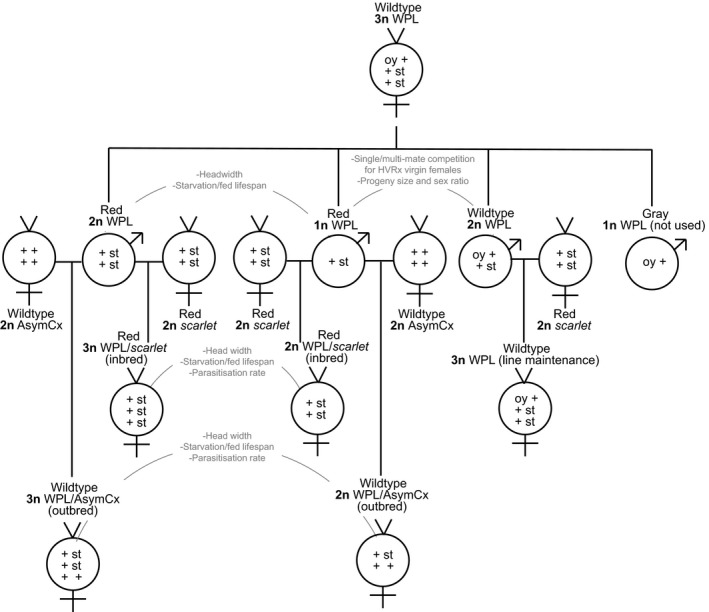
Crossing scheme and individuals used in life‐history trait assays with descriptors of sex, eye color, ploidy level (in bold), and background. Vertical lines indicate descent and horizontal lines indicate a cross. Individuals with Whiting polyploid line (WPL) background have descriptors above their sexual symbols, and unrelated lines used for crossing have descriptors below their sexual symbols. Solid gray arcs indicate non‐polyploid and polyploid counterpart pairings that were compared in life‐history assays. Gray text indicates which life‐history traits were measured in these pairings.

*Nasonia* wasps were cultured at 25 °C, ca. 55% r.h., and L16:D8 light cycle and hosted on commercially produced, purchased *Calliphora* sp. pupae (Titus Blom, Groningen, The Netherlands). The Whiting polyploid line (WPL) originated spontaneously in Whiting's cultures and has been maintained in the laboratory since the 1950s. It was acquired from the John H. Werren laboratory (University of Rochester, Rochester, NY, USA) and has been kept in diapause in our laboratory for 10 years with about one generation of breeding every year. In active culture, the strain is maintained following previously described protocols (Whiting, 1960; Dobson & Tanouye, 1998; Beukeboom & Kamping, 2006). It carries complementary eye color markers *oyster* (*oy*) and *scarlet* (*st*). Homozygotes or hemizygotes for *oy* and *st* have gray and red eyes, respectively, whereas wildtype eyes are dark purple. Wildtype‐eyed triploid females (*oy* +/ + *st/* + *st*) are hosted (are given hosts) as virgins and produce four types of sons: gray‐eyed haploids (*oy* +), red‐eyed haploids (+ *st*), red‐eyed diploids (+ *st*/ + *st*), and wildtype‐eyed diploids (*oy* +*/* + *st*) (Figure 1). About twice as many haploids as diploids are produced, possibly due to embryonic lethality (Whiting, 1960). To continue the line, diploid wildtype‐eyed males (oy+/ + *st*) are mated to virgin (+ *st*/ + *st*) females from the same inbred red‐eye marker stock line used to originate the WPL, *scarlet*. The resultant triploid daughters (*oy* +/ + *st/* + *st*) are used to re‐start the breeding cycle (Beukeboom & Kamping, 2006).

To eliminate possible effects of generation and different eye color markers, we compared life‐history traits of haploid and diploid males in the same generation from the crossing scheme described above. In the male assays for lifespan, progeny size, and offspring sex ratio, only the red‐eyed males were used as these can be haploid or diploid (Figure 1). However, in the mate competition assays, red‐eyed haploids and dark wildtype‐eyed diploids were used to easily distinguish between the ploidy levels. It is possible that red‐eyed males have impaired vision, but a pilot study established that the red‐eye phenotype does not impact the ability of males to acquire mates. For assays comparing females, diploid and triploid females from the same generation were reared on the same host batch to prevent confounding effects of parental age and host quality. To generate these females, red‐eyed haploid (+ *st*) and red‐eyed diploid (+ *st/* + *st*) WPL males of the same generation were mated to virgin females of the mutant *scarlet* line. In a separate experiment to test for effects of inbreeding, these males were also mated to isogenic females of the AsymCx line derived from The Netherlands and cured of *Wolbachia* bacteria (Breeuwer & Werren, 1990; Werren et al, 2010). The WPL/*scarlet* inbred cross (from here on ‘WPL‐inbred’) produced diploid (+ *st/* + *st*) and triploid (+ *st/* + *st/* + *st*) daughters, both with red eyes. The WPL/AsymCx outbred cross (from here on ‘WPL‐outbred’) resulted in diploid (+ *st*/ + +) and triploid (+ *st/* + *st/* + +) daughters, which both have wildtype eyes, and were assessed for the same assays as the WPL. Diploid and triploid daughters within each cross were compared for lifespan and parasitization rate. The individuals used in each assay (polyploid and non‐polyploid counterparts that were compared against each other), their eye marker genotypes and phenotypes, and the crosses used to obtain them are described in Figure 1.

### Ploidy level typing

Ploidy level was determined either through the virgin daughter offspring count method or the flow cytometry method. The virgin daughter method was used for lifespan assays because the flow cytometry method requires the male to be freshly killed. For the virgin daughter offspring count method, a WPL male was given 24 h to mate with a virgin AsymCx female and the female was subsequently provided with two hosts. A pilot study established that there is no lifespan difference between males mated through this method and unmated males. Approximately 2 weeks later, daughters were collected as black pupae and allowed to eclose. A virgin daughter of each cross was given two hosts and their offspring were allowed to develop to adulthood and emerge from hosts naturally. Triploids have low fecundity due to egg aneuploidy (given that *Nasonia* have five chromosomes, approximately one out of 32 eggs are euploid and viable). A pilot study found that scarlet virgin diploid females produce on average 29.00 ± 25.68 (mean ± SD; n = 45) offspring with two hosts, but virgin triploid WPL females produce only 2.60 ± 2.35 offspring (n = 40) (with the maximum being nine). Therefore, in this study, if fewer than eight offspring from the two hosts emerged after 2 weeks, the female was assigned triploid status and her experimental father was assigned diploid status. If more than 15 offspring emerged, the female was assigned diploid status and her father was considered a haploid male. Those females that had 9–15 offspring could not be unambiguously typed for ploidy. This applied to <5% of females and they were excluded from analyses.

The flow cytometry method was used to type the ploidy of males in the progeny size and progeny sex ratio assays, one trial of the mate competitions, and the daughters of the mate competitions. It was adapted from previous polyploid wasp studies (Beukeboom et al., 2007; Thiel et al., 2014). Heads of wasps were removed with a razorblade and stored at −20 °C in individual 1.5‐ml Eppendorf tubes. Only heads were used for flow cytometry as ganglial cells have consistent ploidy but body cells can be endopolyploid (Fankhauser, 1945; Wertheim et al., 2013). Upon preparation for flow cytometry, each head was ground for 30 s with an electric VWR pellet mixer in 500 μl ice‐cold Galbraith buffer [21 mm MgCl_2,_ 30 mm tri‐sodium citrate dehydrate, 20 mm 3‐[N‐morpholino] propane sulfonic acid (= MOPS), 0.1% Triton X‐100, and 1 mg l^−1^ RNase A]. Each sample was then poured through a Falcon 5‐ml tube with a cell strainer cap to remove exoskeletal material from the cell solute. Twenty μg of propidium iodide (10 μl of a 2.5 mg ml^−1^ solution) (Sigma‐Aldrich, St. Louis, MO, USA) was added to each sample, and the tube gently flicked to mix. Samples were put on ice and immediately run through a BDFACS ARIA II flow cytometer. Individuals were assigned ploidy based on how their signal matched those of reference 1n male (AsymCx), 2n female (AsymCx), and 3n female (WPL females) samples. Individuals that had an ambiguous FACS reading (e.g., a large amount of debris) were excluded from analyses (ca. 10% of samples).

### Lifespan

Lifespan was measured for the WPL red‐eyed males (haploid and diploid) and the WPL‐inbred and WPL‐outbred females (diploid and triploid). Following parasitization, cultures were checked for emerged offspring every day starting at day 10 after oviposition. Upon emergence of the first wasp, host pupae were opened and all individuals were collected. Only individuals fully eclosed from the pupal case and free walking were used for lifespan assays. Each individual was housed in a 63 × 11 mm tube and returned to standard rearing conditions. Every 12 h, all tubes were checked for dead wasps. A wasp was scored as dead if it was found at the bottom of the tube and was unresponsive to gentle prodding with a fine‐tip brush. These assays were conducted under both starvation and feeding conditions. Under feeding conditions, a strip of filter paper dipped in 10% sucrose solution was placed into the tube and replaced every 3 days.

### Male mate competition

Male mate competition assays were conducted to compare haploid vs. diploid male ability to acquire female mates, for both single‐ and multiple‐mate availability scenarios. From a hosting of virgin female triploid WPL, males were collected and sorted by eye color. In each mate competition setup, a red‐eyed male (presumed haploid, as about 75% of red‐eyed males are haploid; Whiting, 1960) and a wildtype‐eyed male (diploid), randomly chosen and less than 1 day old, were placed in a 63 × 11 mm tube. Whereas red‐eyed haploid and red‐eyed diploid competition pairs would have been ideal to exclude effect of eye color, the probability of randomly chosen pairs being one haploid and one diploid was too low. In contrast, the diploidy of males with wildtype eyes is known a priori. Red‐eyed haploids instead of oyster‐eyed haploids were used because too few of the latter were produced. In a pilot study using only haploid males, females did not discriminate against oyster‐eyed (from the pure *oyster* line) or red‐eyed (from the pure *scarlet* line) males when given a choice between an eye‐color mutant or a wildtype‐eyed AsymCx male. Therefore, eye color is not likely to be a factor in male mate competition success or biased results here.

In the male mate competition setups, 1‐day‐old virgin female mates were used from the genetically variable laboratory population HVRx (van de Zande et al., 2014). This population was chosen to better test male ability to attract mates of genetically variable backgrounds, and to avoid female re‐mating behavior. Female polyandry evolves in inbred and long‐established laboratory lines (van den Assem & Jachmann, 1999; Burton‐Chellew et al., 2007; Shuker et al., 2007), but the HVRx population is recently derived from wild populations, maintained to prevent selection for laboratory traits, and has not been bred beyond 150 generations. Thus, females of this population are likely to be strongly monandrous like wild *Nasonia* (van den Assem et al., 1980; Burton‐Chellew et al., 2007; Grillenberger et al., 2008). For single‐mate competition, one HVRx virgin female was provided to the competing haploid male and diploid male (n = 22 trials), and in the multiple‐mate competition setups, 10 HVRx virgin females were provided (n = 20 trials). All setups were given 24 h to mate. Afterwards, all males were frozen and stored at −20 °C.

Female mates were hosted individually and after 2 weeks, all offspring were collected and the larval (immature), male, and female offspring were counted for each mate. Total family size (including larvae) and offspring sex ratio (disregarding the few larvae, as they cannot be sexed) were recorded. Offspring were then stored at −20 °C for the follow‐up flow cytometry analysis, using a single daughter of each female. If a daughter was typed as diploid, it was assumed that this was the ploidy of all her sisters, and that her father (the female's mate) was the haploid red‐eye male. Conversely, if a daughter was typed triploid, it was assumed that she and all her sisters were sired by the diploid dark‐eyed male. In the unlikely case that multiple‐sired progeny would have occurred, the chance to score it as fathered by a haploid or diploid male would have been equal, so this would not bias the results. Females that produced all‐male offspring were scored as not mated and discarded from the analysis. Multiple‐mate trials in which a majority of the females did not mate were also discarded. If all mated females of a multiple‐mate trial were found to have mated with a diploid mate, this was interpreted to mean that it was possible that the red‐eyed male and the wildtype‐eyed male were both diploid as red‐eyed males can also be diploid. In the single case in which this occurred, to confirm that a haploid male vs. a diploid male competition took place, the frozen red‐eyed male was processed with flow cytometry and its haploid status was verified.

### Progeny size and sex ratio

Although only female offspring reflect a male sire, signals from male mates may potentially influence female decisions about total family size and offspring sex ratio. Therefore, the total number of offspring (progeny size, including both males and females) and offspring sex ratio (proportion males out of total offspring) data were collected for the AsymCx female mates of WPL red‐eyed haploid and red‐eyed diploid males used in lifespan assays. The same data were collected for HVRx females in the mate competition trials that used haploid red‐eyed males and diploid wildtyped‐eyed males.

### Female parasitization rate

Diploid and triploid virgin females of the WPL‐inbred and WPL‐outbred lines were tested for their relative ability to parasitize an excess of hosts. One day post pupal eclosion, each female was given a first set of 10 hosts in a 63 × 11 mm tube, kept under standard conditions. Every 2 days, they were each transferred to another 10 fresh hosts, and the previous hosting sets were allowed to develop. This was repeated for the entirety of the specimen's lifetime. After 2 weeks the hosts were scored for fly or wasp emergences. If a fly emerged, it was scored as a failed parasitization. If neither a fly nor wasps emerged it was scored as parasitized (i.e., stung by the wasp with or without oviposition). It is possible that for some of these hosts, non‐emergence could be attributed to bad host quality rather than parasitization, but the proportion of bad‐quality hosts in our culturing is typically very low (<5%) and considered to have negligible effect on results. Deaths were also recorded every time females were re‐hosted, to measure approximate lifespan.

### Head width (body size)

Head width data were collected to infer whether body size differs between non‐polyploids and polyploids, and whether it is a factor in any significant differences for life‐history trait phenotypes as is often the case with insects (Beukeboom, 2018). Whereas gregarious parasitoids such as *Nasonia* spp. can vary greatly in size based on host effect (e.g., genetically identical males can vary by a factor of 2; Groothuis & Smid, 2017), the host batch was the same for directly compared datasets and host effect on body size was assumed to be insignificant due to random use. As the abdomen size can fluctuate over a specimen's lifetime from feeding or egg load, head width was used as a proxy for total body size (as in, e.g., Charnov & Skinner, 1984). Head width was measured for a subset of haploid and diploid males of the WPL, and a subset of diploid females and triploid females of the Whiting inbred and Whiting outbred lines. Measurements were taken of heads separated from bodies at 5× magnification with a Carl Zeiss Stemi 508 dissection microscope with a W‐PI 10×/23 eyepiece and 14 mm reticule.

### Statistical analysis

Statistical tests were performed in SPSS Statistics v.25 (IBM, 2017) and RStudio v.1.0.153 (R Core Team, 2014) with significance level α = 0.05. Shapiro–Wilk tests and Levene tests were used to test for normality and homogeneity of variance for all datasets. A two‐sample t‐test was used if data were normally distributed and had equal variance. A Welch t‐test was used if distributions were normal but variances were unequal. Mann–Whitney U tests were used if data were neither normal nor equal in variance. A binomial test was used to test for significant difference in the mate competition ability of haploids vs. diploid males, with the null hypothesis (H_0_) that haploid males and diploid males have equal mate competition ability (each mated with half of the females for both the individual‐ and multiple‐mate trials). Additionally, a general linear mixed model was used to test whether females were more likely to mate with a haploid or a diploid male in the single‐mate and multiple‐mate experiments. This used a multinomial logistic regression with a generalized logit link, with trial number set as a random effect. A Satterthwaite approximation and an estimation of robust variance were used to account for non‐normality and low sample size. Survival graphs were generated for all lifespan datasets of corresponding non‐polyploids and polyploids (for starvation, feeding, and female parasitization rate) and analyzed with the log‐rank (Mantel‐Cox) test to compare survival distributions. To test for diploid vs. triploid parasitization ability, a binomial general linearized mixed model was used for the number of hosts parasitized per host set using a binary logistic regression link. For this, day and individual were set as random effects, and ploidy state (diploid or triploid) as a fixed effect. Again, a Satterthwaite approximation and an estimation of robust variance were used. For brevity, most data means, sample sizes, and P‐values of tests are reported in Table 1. The general linearized models and binomial general mixed models are reported in the Supporting Information.

**Table 1:** Data summary of life‐history assays. Unless otherwise noted, data are means (± SD) of traits for non‐polyploids (male haploids, female diploids) and polyploids (male diploids, female triploids) of the Whiting polyploid line (WPL)‐inbred and WPL‐outbred backgrounds. Assays with significant differences (P<0.05) between the non‐polyploid and polyploids are indicated with an asterisk

Trait	Assay	Non‐polyploid (n)	Polyploid (n)	P	Test
Lifespan (starved) (days)	WPL‐inbred male	5.66 ± 2.82 (126)	5.75 ± 1.88 (20)	0.34	Mann–Whitney U
	WPL‐inbred female	5.61 ± 1.05 (42)	5.25 ± 1.13 (12)	0.48	Mann–Whitney U
	WPL‐outbred female*	3.46 ± 0.89 (80)	3.78 ± 0.79 (67)	0.037	Mann–Whitney U
Lifespan (fed 10% sucrose solution) (days)	WPL‐inbred male	22.21 ± 8.79 (71)	23.33 ± 10.55 (20)	0.78	Mann–Whitney U
	WPL‐inbred female	12.37 ± 1.13 (41)	12.59 ± 9 (12)	0.51	Mann–Whitney U
	WPL‐outbred female*	14.05 ± 9.09 (228)	12.39 ± 7.22 (114)	0.009	Mann–Whitney U
Male mate competition (trials won)	WPL‐inbred male (single competition)	11	7	0.48	Binomial
	WPL‐inbred male (multi competition) (3 ties)	6	6	0.78	Binomial
Progeny size (total, male and female)	WPL‐inbred male (no choice)	49.30 ± 13.15 (56)	49.26 ± 8.18 (23)	0.99	Welch's t‐test
	WPL‐inbred male (single competition)	54.18 ± 10.2 (11)	49.42 ± 8.43 (7)	0.35	Two sample t‐test
	WPL‐inbred male (multi competition)	54.97 ± 14.63 (71)	55.07 ± 15.57 (59)	0.97	Two sample t‐test
Progeny sex ratio (male/total)	WPL‐inbred male (no choice	0.147 ± 0.08 (56)	0.181 ± 0.11 (23)	0.16	Mann–Whitney U
	WPL‐inbred male (single competition)	0.33 ± 0.25 (11)	0.38 ± 0.22 (7)	0.82	Mann–Whitney U
	WPL‐inbred male (multi competition)*	0.29 ± 0.15 (71)	0.50 ± 0.23 (59)	<0.001	Mann–Whitney U
Female parasitization lifespan (days)	WPL‐inbred female*	14.13 ± 3.79 (30)	10.92 ± 4.39 (13)	0.05	Mann–Whitney U
	WPL‐outbred female*	19.98 ± 6.35 (47)	15.17 ± 6.35 (36)	<0.001	Mann–Whitney U
Female parasitization (total hosts parasitized)	WPL‐inbred female*	49.07 ± 15.14 (30)	26 ± 16 (13)	<0.001	Two sample t‐test
	WPL‐outbred female*	80.79 ± 26.75 (47)	40.56 ± 24.08 (36)	<0.001	Mann–Whitney U
Female parasitization (% hosts parasitized out of total offered)	WPL‐inbred female*	69 ± 13 (30)	44 ± 19 (13)	<0.001	Mann–Whitney U
	WPL‐outbred female*	79 ± 14 (47)	53 ± 20 (36)	<0.001	Mann–Whitney U
Head width (mm)	WPL‐inbred male*	0.699 ± 0.030 (56)	0.732 ± 0.049 (23)	<0.001	Mann–Whitney U
	WPL‐inbred female*	0.686 ± 0.059 (50)	0.768 ± 0.052 (36)	<0.001	Mann–Whitney U
	WPL‐outbred female	0.804 ± 0.0563 (78)	0.795 ± 0.067 (80)	0.43	Welch's t‐test

## Results

### Lifespan

**Figure 2:**
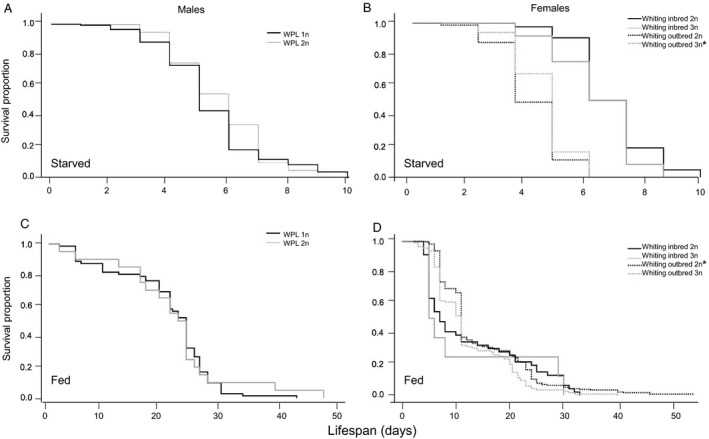
Survival curves (proportion of individuals still alive over time) of non‐polyploid and polyploid (A) starved males, (B) starved females, (C) fed males, and (D) fed females in the inbred and outbred Whiting polyploid line (WPL). Males assayed were inbred red‐eyed haploids and red‐eyed diploids, females assayed were diploids and triploids. An asterisk denotes a significantly longer lifespan than the counterpart. Note the different scales on the horizontal axes.

Polyploids generally do not live longer or shorter than non‐polyploids. This is true for diploid males and triploid females of the Whiting inbred line, but not the triploid Whiting outbred line females (Figure 2). The average lifespan of starved WPL‐inbred red‐eyed haploid males was similar to that of the red‐eyed diploids (Table 1), and their survival distributions were similar (log‐rank test: χ^2^ = 0.167, d.f. = 1, P = 0.68) (Figure 2A). When fed, there was also no difference in lifespan between red‐eyed haploid and diploid males (Table 1) and survival distributions were similar (log‐rank test: χ^2^ = 0.050, d.f. = 1, P = 0.82) (Figure 2C). As body size (i.e., head width) was not significantly different between haploid and diploid males (see below), body size differences did not factor in these results.

Under starvation conditions, diploid WPL‐inbred females had the same lifespan as triploid WPL‐inbred females (Table 1), and survival distribution was also the same (log‐rank test: χ^2^ = 0.856, d.f. = 1, P = 0.36) (Figure 2B). When fed, the average lifespan of diploids and triploid females of this line again did not differ (Table 1) nor were their survival distributions different (log‐rank test: χ^2^ = 0.801, d.f. = 1, P = 0.65) (Figure 2D). Starved diploid WPL‐outbred females lived 0.32 days less than triploid WPL‐outbred females (Figure 2B). This difference, although small, is significant (Table 1), as is the difference in survival distribution (log‐rank test: χ^2^ = 4.231, d.f. = 1, P = 0.04) (Figure 2B). In the fed assay, WPL‐outbred diploid females lived a significant 2.1 days longer than WPL‐outbred triploids (Table 1, Figure 2D) and survival distributions are significantly different (log‐rank test: χ^2^ = 9.035, d.f. = 1, P = 0.003) (Figure 2D). Thus, there were no significant lifespan differences in the original WPL‐inbred genetic background for either sex. However, in the outbred WPL background polyploid females lived slightly longer than the non‐polyploids under starvation conditions, but under fed conditions, they lived more than 2 days shorter than the non‐polyploids.

### Male mate competition

There is no difference in mate competition ability between haploid and diploid males for either single female or multiple female mates. In the single‐mate competition assay, in 18 out of 22 trials, the HVRx female mated with a male and produced daughters (the other four trials resulted in unmated virgins that produced only sons). Of these 18 successful trials, the female mated with the haploid male in 11 trials and with the diploid male in seven trials, which is not a significant difference (Table 1).

In the multiple‐mate competition assays, for 15 out of 20 trials, more than five out of the 10 females mated and produced female offspring. Of these 15 trials, in six trials the haploid male mated with a majority of the females, in six trials the diploid mated with a majority of the females, and in three trials the haploid male and the diploid male mated with the same number of females (tied). Regardless of whether the diploid or haploid male acquired more mates in a specific trial, mating competition success ranged widely for each male (i.e., 0–100% for haploids, 0–100% for diploids) (Table S1). In one multiple‐mate competition trial, all females were found to have mated with a diploid male. As a low proportion of WPL red‐eyed males are diploid, it was possible that the red‐eyed male used in this trial as presumed haploid competitor was actually diploid. Flow cytometry was used post‐hoc to assess the ploidy of the red‐eyed male. It was confirmed a haploid; therefore, this trial was retained in the analysis.

A binomial general linearized model was used to test whether females were more likely to mate to a haploid male or a diploid male. For the single‐mate competition, the 18 females were not more likely to mate with either type of male, and the same applies for the 114 females of the multiple‐mate competition (Table S2). Taken together, although sample sizes are limited, these results do not point towards any difference in male mate competition ability between ploidy levels.

### Progeny size and sex ratio

**Figure 3:**
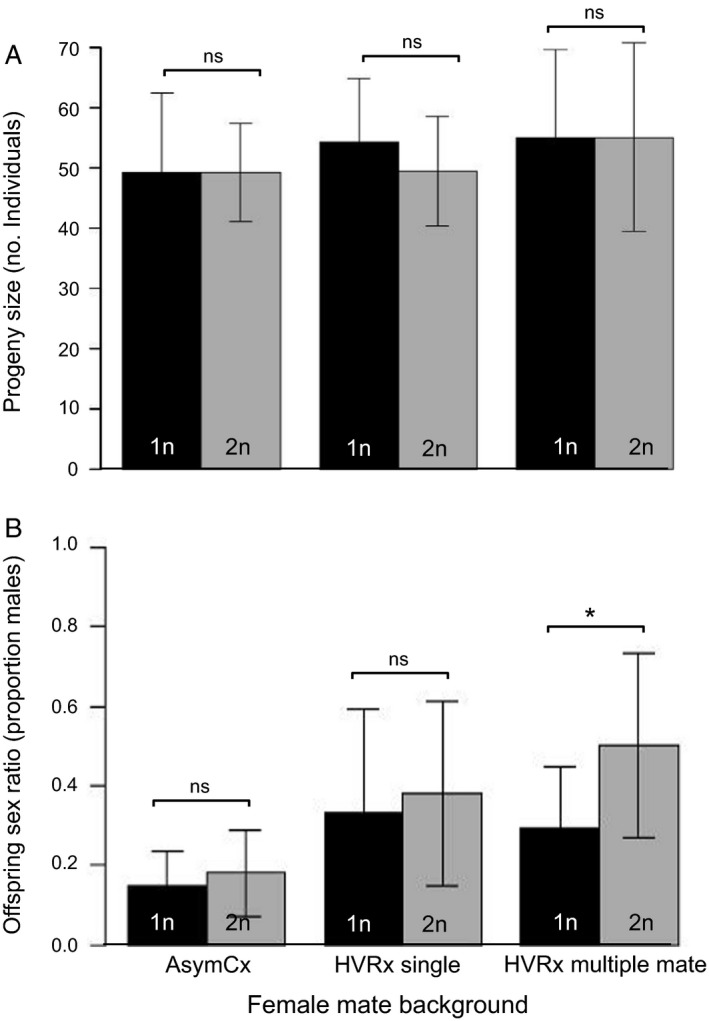
Mean (± SD) (A) progeny size (no. family members) and (B) offspring sex ratio (proportion sons) of haploid (1n) and diploid (2n) Whiting inbred polyploid line males with AsymCx and HVRx female mates in the single‐ and multiple‐mate competitions. The asterisk denotes a significant difference between haploid vs. diploid fathers (Mann–Whitney U test: P<0.0001; ns, P>0.05).

Diploid males do not sire more or fewer offspring than haploid males. This applies for red‐eyed males mated to females in a no‐choice experiment and those in single‐female and multiple‐female mate competition assays. In the no‐choice experiment, a WPL haploid red‐eyed male and a WPL diploid red‐eyed male mated to an AsymCx female had the same total progeny size (male and female offspring of the female mate) and offspring sex ratio (males/total) (Table 1, Figure 3).

In the single‐mate competitions, an HVRx female mated with either a WPL red‐eyed haploid male or a WPL wildtype‐eyed diploid male. In 11 out of 18 trials, the female mated with the haploid male. In the remaining seven trials, the female mated with the diploid wildtype‐eyed male. They did not significantly differ in progeny size or offspring sex ratio (Table 1). In a multiple‐partner experiment 10 HVRx females could be mated by a WPL red‐eyed haploid male or WPL wildtype‐eyed diploid male. Including the females that were not used in mate competition analyses because a majority of females in their trial did not mate, a total of 130 females mated, 71 with the haploid male, and 59 with the diploid male (Table S1). Progeny size is not significantly different between haploids and diploids, but offspring sex ratio was significantly more male‐biased for diploid males (Table 1). For the multiple‐partner results the higher male offspring sex ratio for females that mated with a diploid male is unusual, as it is typical for *Nasonia* spp. females to produce highly female‐biased broods under favorable conditions (Whiting, 1967).

### Female parasitization rate

**Figure 4:**
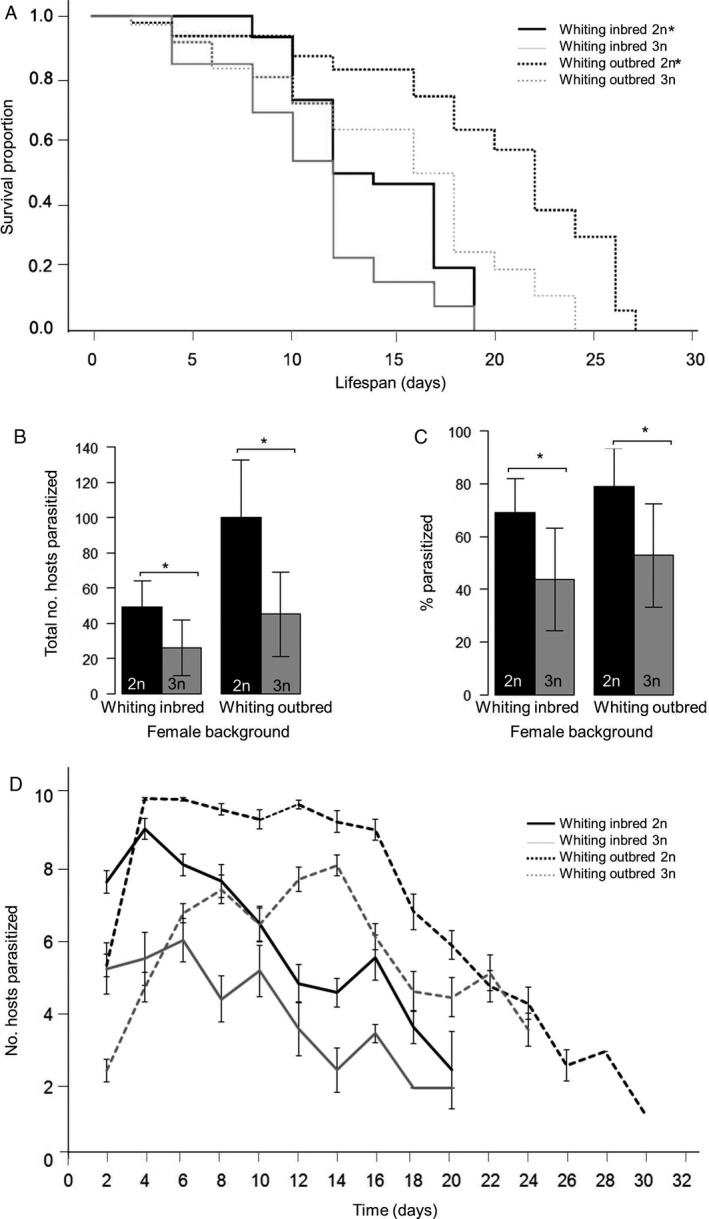
Female parasitization rate of diploids (2n) and triploids (3n) of the Whiting inbred and the Whiting outbred backgrounds: (A) survival curve (proportion of individuals still alive over time), and mean (± SD) (B) total number of hosts parasitized, (C) percentage of total hosts offered parasitized, and (D) number of hosts parasitized for each set of 10 fresh hosts offered every 2 days until death. An asterisk denotes a significant difference (in panel A it denotes the significantly longer‐lived counterpart) (Mann–Whitney U test: P<0.05).

In the parasitization rate assay, WPL females were offered 10 fresh hosts every 2 days until death. Triploid females lived significantly shorter and parasitized significantly fewer hosts over their lifetime than diploid females (Figure 4). For the WPL‐inbred females, diploid females lived on average a significant 3.21 days longer then triploids (Table 1). For the WPL‐outbred females, diploid females lived on average a significant 4.81 days longer (Table 1). Survival distributions were also significantly different between triploids and diploids for both backgrounds, with a greater fraction of diploid females surviving at every time point (log‐rank test: χ^2^ = 12.249, d.f. = 1, P<0.001) (Figure 4A). Thus, when given a continuous supply of hosts, used by the female both for oviposition and as a food source, triploid females live significantly shorter than diploid females. This contrasts somewhat with the results of the ‘fed lifespan’ assay in which females was given 10% sucrose solution, as there was no difference between WPL‐inbred diploid and triploid females, but WPL‐outbred triploid females lived longer than diploid females (Figure 2D).

The diploid WPL‐inbred females parasitized almost twice as many hosts as their triploid counterparts, with a parasitization rate (host parasitized/hosts offered) that was a significant 25% higher (Table 1). The diploid WPL‐outbred females also parasitized about double the number of hosts relative to the outbred triploids, with a 26% higher parasitization rate (Figures 4B and 4D). Interestingly, although the inbred and outbred backgrounds cannot be directly compared as independent experiments, the outbred background had much higher parasitization success overall compared to the inbred background (for both the diploid and triploid females) (Table 1).

A binomial general linearized mixed model was used to analyze the relationship between ploidy and parasitization success (Table S3). For all backgrounds, parasitization ability declines over time as the female ages (Figure 4D), but for the Whiting inbred background, controlling for day (host set) and the individual, the diploid is 4.3× more likely to parasitize any given host than the triploid; the diploid WPL‐outbred female is 7× more likely to parasitize a host than the triploid WPL‐outbred female (Table S3).

### Body size

Head width, used as proxy measure for total body size, differed significantly between non‐polyploid and polyploid individuals for two out of three cases assessed. For WPL males, diploids are larger than haploids (Table 1). For the WPL‐inbred, diploid females are smaller than triploids (Table 1). In contrast, diploid and triploid females of the WPL‐outbred are of similar size (Table 1).

## Discussion

The consequences of inbreeding and polyploidy are well known in CSD‐species because of the sterile diploid male vortex, but polyploid phenotypes in non‐CSD species have not been studied. In assaying a range of life‐history traits in a polyploid line of the non‐CSD parasitoid *N. vitripennis*, there appear to be a few disadvantages related to the polyploid state (reproductive impairment, reduced parasitization). These results can be interpreted for their effects on the performance of *N. vitripennis* as a biocontrol agent, as it is used for muscid pest control in livestock rearing (Kaufman et al., 2001a, 2001b; Skovgård & Nachman, 2017), albeit uncommonly. But more importantly, the results of this model species can be used to judge the possible pros and cons of using polyploids in biocontrol programs.

Lifespans of *N. vitripennis* WPL haploid and diploid males (under both starved and fed conditions) are equal, similar to results found for other hymenopteran species such as CSD‐species *Habrobracon hebetor* (Say), a parasitoid wasp (Clark & Rubin, 1961), and the ant *Hypoponera opacior* (Forel) (Kureck et al., 2013). This suggests that hymenopteran diploid males are not impaired in longevity compared to their haploid counterparts whether they have CSD or not. For both ‘starved and fed lifespan’ assays, triploid females lived as long as diploid females of the WPL‐inbred line, but diploid females of the WPL‐outbred line lived longer than the triploids. The reason for this difference between backgrounds is unknown. It may be due to co‐adapted gene complexes being unbalanced in the WPL‐outbred cross relative to the WPL‐inbred cross, which would imply outbreeding depression. A shorter lifespan may not necessarily be disadvantageous for individual fitness or a biocontrol program if critical functions are concentrated in early life. For example, if fecundity peaks early and reproductive output and parasitization ability is negligible by the end of life, then a shorter lifespan may not carry large fitness disadvantages. Notably, the WPL‐inbred background may confer a greater degree of starvation resistance than the WPL‐outbred, as both WPL‐inbred diploid and triploid females lived longer than their WPL‐outbred counterparts, a trend not observed under fed conditions.

In both single‐ and multiple‐mate competitions for females of the HVRx background—a genetically variable and recently derived laboratory population (van de Zande et al., 2014) chosen specifically to circumvent multiple mating, as wild outbred *Nasonia* spp. are strongly monandrous (van den Assem et al., 1980; Burton‐Chellew et al., 2007; Grillenberger et al., 2008)—, WPL haploids and diploid males were equally successful in acquiring mates. However, the small sample sizes and low statistical power of this dataset should be noted. Owing to the labor intensiveness of this assay and the need to discard trials in which females do not mate, a larger sample size was not possible. However, we do not think that additional trials would change results. In the *Nasonia* system, males present courtship behaviors to females, which then have control over mate acceptance. Although the males of this study were not evaluated for any specific differences in courtship, the results imply that they may produce similar olfactory, visual, and behavioral cues that are not discriminated against by the females. Alternatively, differences may exist and went unobserved, but did not ultimately change the attractiveness of the diploid male. This would be similar to, for example, the CSD‐species *Cotesia glomerata* (L.), in which diploid males initiate courtship sooner than haploids, but have neither higher nor lower mating success (Elias et al., 2009). The success of diploid males in this study matches results for haploid and diploid male mate competitions of other CSD‐parasitoid wasp species (reviewed by Harpur et al., 2013; Thiel et al., 2014). This implies that the utility of non‐CSD diploid males in breeding programs would not be hampered by unattractiveness, as even in the presence of conventional haploid males, females are receptive to mating with diploids.

When WPL haploid and diploid males were given females of identical genetic background (AsymCx) in a scenario in which there were no competing males, their family size and offspring sex ratio were the same. The offspring sex ratio of those females that mated with the WPL haploid and diploid males in no‐choice assays were approximately 0.15, which is not highly divergent from the 0.10 proportion that is possible when mated females have ideal conditions (i.e., low competition, high host quality) (Whiting, 1967). The diploid males of the WPL line are thus exceptionally fertile relative to the near‐universal infertility of CSD diploid males (van Wilgenburg et al., 2006; Heimpel & de Boer, 2008; Elias et al., 2009; Harpur et al., 2013), although exceptions exist for the solitary vespid wasp *Euodynerus foraminatus* (de Saussure) (Cowan & Stahlhut, 2004), the ichneumonid wasp *Diadromus pulchellus* Wesmael (El Agoze et al., 1994), and braconid parasitoids *Cotesia vestalis* (Haliday) (de Boer et al., 2007) and *C. glomerata* (Elias et al., 2009). These CSD species with fertile diploid males are capable of producing daughters. The first two have haploid sperm and produce diploid females, and the *Cotesia* spp. have diploid sperm and produce triploid females. In at least one other non‐CSD species (the braconid wasp *Asobara japonica* Belokobylskij) diploid males are also capable of producing many triploid daughters, although they do not sire as many offspring as haploids (Ma et al., 2015). This may suggest that male diploids in the non‐CSD class have higher fertility than CSD‐species in general. Interestingly, whereas progeny size was also similar between the WPL haploid and diploid male in the mate competitions, offspring sex ratio of diploid males was higher in the mate competition using genetically variable HVRx females (0.503, compared to 0.293 of haploid males).

Possible explanations for higher sex ratios among progenies sired by diploid males are lower sperm count in diploid males or impaired fertilization capacity of diploid sperm. It was previously shown that *N. vitripennis* haploid males are able to mate with 13–18 females in rapid succession before being depleted of sperm (Beukeboom, 1994; Chirault et al., 2016). These studies were the basis for deciding to provide the males with 10 potential female mates. It is possible that diploid males have fewer sperm and experienced faster sperm depletion with successive matings in the multiple‐mate trials. Corroborating this, in a study of haploid males, the most drastic reduction in sperm transfer followed the first mating (Chirault et al., 2016), which may explain why offspring sex ratio differences are not observed in the no‐choice matings and single‐mate competitions. Diploid sperm may also be lower in quality; for example, diploid sperm of CSD *Habrobracon* wasps apparently fail to penetrate eggs (MacBride, 1946). The results of this study are similar to those of other parasitoid wasps (CSD braconids) where females did not discriminate against diploid males as mates, but produced fewer daughters (Elias et al., 2009; Thiel et al., 2014). Regardless of why the male proportion is so high in progenies of diploid males in the multiple‐mate competition experiments, this result suggests that diploid males of both CSD and non‐CSD species will have lower lifetime fitness even if, as is the case with the WPL line, they live as long as haploids and have high reproductive success with first mates. This may severely reduce their usefulness in breeding programs, but the degree of difference between diploid and haploid lifetime fecundity has not been rigorously tested. Follow‐up investigation is needed to compare *N. vitripennis* haploid and diploid male for lifetime fitness, and to examine diploid sperm itself for impairment.

To the authors’ knowledge, the parasitization ability of triploid female parasitoid wasps has not been tested for any species previously, possibly because of their rarity. Therefore, the WPL gives first insight on the parasitization ability of polyploid parasitoid females. Female *Nasonia* are synovigenic, meaning that they emerge from their host with a partial complement of mature eggs and can reproduce right away, but will continue to produce eggs as long as they consume enough protein (Pannebakker et al., 2013). Perhaps reflecting the better overall nutritional composition of host hemolymph vs. the sucrose solution of the ‘fed lifespan’ assays, females generally lived longer in this assay (the exception being the WPL‐diploids). This matches observations of another chalcidoid wasp that is possibly non‐CSD, *Aphytis melinus* DeBach, living longer and being more fecund with additional host feeding vs. sugar feeding alone (Heimpel et al., 1997). However, the parasitization assay of this study shows that female triploids both have shorter lives and are inferior lifetime parasitizers relative to diploids even with unlimited resources. The hypothesis that triploids could be as proficient at killing hosts as diploids despite their low fecundity was based on how *Nasonia* venom efficiently induces mortality in hosts, even in the absence of any parasitoid feeding (Rivers et al., 1993). As there was no evidence to suggest that the venom of triploid females is attenuated, we predicted that host killing ability might be retained. However, triploids of both the WPL‐inbred and WPL‐outbred had poor parasitization performance in comparison to their diploid counterparts. They parasitized far fewer hosts overall and a lower percentage of offered hosts per set.

The underlying cause of reduced parasitization ability in triploid females is not clear, but may possibly be attributed to reduced fecundity. A large number of the triploid's offspring die in the egg stage as aneuploids, so it is possible that more hosts survived because of fewer offspring surviving to larval stage to consume hosts. Somewhat supporting this, while parasitization gradually declined over the lifespan of both diploids and triploids, parasitization would increase and decrease with alternate host sets. This sinusoidal parasitization success pattern matches the pattern expected for periodic egg depletion and replenishment as is typical of female *Nasonia* synovigenic life‐history strategy, suggesting that every other host set may have had more living offspring to kill hosts. Regrettably, as host killing was the focus of this assay, offspring were not counted. Alternatively, it may be that triploids simply did not sting as many hosts, and therefore failed to transfer venom as often, or that they did not do as much host feeding as diploids, which is also a factor in host killing (Kidd & Jervis, 1989). Future studies should investigate these behaviors in triploids as possible explanatory factors for parasitization rate deficiency, but these results suggest that the triploid state is a major impediment to biological control efficiency.

Notably, an outbreeding advantage for parasitization may have been observed in the females of this study. The WPL‐inbred cross has been used to maintain the line for decades, so the cross with AsymCx females could be considered a single generation of outcrossing. Although the assays for inbred and outbred females were run as separate experiments, compared to their WPL‐inbred counterparts, outbred diploid and triploid females parasitized more hosts over their lifetime and parasitized a higher percentage of hosts. Impressively, the triploid outbred females parasitized more than double the total number of hosts parasitized by the inbred triploids (although they still parasitized only half as many hosts as the outbred diploids). Outbred lifespans were also approximately a third longer. Lifespan extension with outbreeding is consistent with the observation of Luna & Hawkins (2004), who found that outbreeding can improve fecundity and lifespan in *N. vitripennis*. These differences are too large to be accounted for by any slight variation in host batch quality. Higher parasitization rate overall for the WPL‐inbred line could have been from a larger number of offspring surviving to larval stage to feed on the host, in which case the poor parasitization ability of inbred triploids was partly ‘rescued’ by outbreeding. Unfortunately, offspring data were not collected and compared between the outbred and inbred females because low and similar fecundity from aneuploidy was expected for both, and should be considered in future studies of triploid female performance.

Body size is an important factor in the overall fitness of an insect. In general, larger insects outcompete smaller conspecifics in reproductive output and resource competition (Beukeboom, 2018). In this study, the polyploid is either not significantly different in body size from the non‐polyploid, or is only slightly larger. This is consistent with studies on other invertebrates (reviewed by Fankhauser, 1945) and fits the overall trend for Hymenoptera (A Thiel, pers. comm.). The diploid *N. vitripennis* WPL males were slightly larger than the haploids according to the head width proxy measurement, albeit with a large overlap in values. This size difference did not affect any of the life‐history traits assayed in this study. Similarly, in females, in one background (WPL‐outbred) the triploid females were similarly sized as the diploids, and in the other background (WPL‐inbred), triploids were significantly larger. The reason for this incongruency between backgrounds is not known. It is possible that polyploidy in itself does not cause major changes in body size for females, but is a consequence of the *scarlet* mutant background used to maintain the WPL. Related to this, the WPL‐outbred cross could have counteracted an inbreeding effect on size within the *scarlet* background. Both diploids and triploids of this background are bigger than those of the inbred background. This suggests that outbreeding can be used to increase polyploid size, but the results here do not indicate any practical advantage to doing so.

The results of this study can be interpreted in the context of the use of non‐CSD parasitoids for biological control. As polyploidy may be more viable in non‐CSD species, this suggests that advantageous polyploid applications in non‐CSD parasitoid wasp breeding may be more feasible than for CSD‐species. It can, for example, hypothetically assist with sex‐specific tradeoffs, where traits beneficial to females but detrimental to males would be purged in the haploid state but be masked in the diploid state, meaning that it can be passed on to the next generation of females (the more important sex to biological control). Our results do not support a promising role of polyploidy in hymenopteran breeding, although there may be some remedies to some of these drawbacks and ways to improve the use of polyploids.

For possible diploid male fecundity loss from fewer or less functional sperm, the number of diploid males can be maximized to increase the net number of polyploid females in the next generation. In *Nasonia* the development of normal diploid embryos can be switched from female to male by silencing feminizing gene elements (Verhulst, 2010). This can be exploited to produce families that consist predominantly of diploid males. For females, the problem of poor parasitization is harder to resolve, but the proposed outbreeding solution can be tested in the future to explore to what extent their biocontrol performance can be optimized. In addition, artificial selection may be applied to increase diploid male reproductive output and triploid female parasitization ability. This study, however, indicates a need for broader study of polyploid phenotypes of non‐CSD species, the problems that they can manifest, and the specific solutions needed.

## Supporting information


**Table S1.** Winners of single‐ and multiple‐mate competitions of Whiting inbred lines for HVRx females. Grayed‐out trials were discarded from analyses because the female of single competition did not mate, or a majority of females in a multiple‐mate competition did not
**Table S2.** Results from the general linearized model for likelihood of females to mate with a haploid or diploid male in the multiple‐mate experiment. The intercept indicates the model incorporating trial number as a random factor. The reference category is set to the haploid male
**Table S3.** Results from the binomial general linearized mixed model for parasitization rate of inbred and outbred Whiting line females. The intercept indicates the model incorporating random factors ‘day’ (host set) and ‘individual’, and the fixed effect ‘ploidy’ or ‘background’. The data for the diploid are relative to the triploid (0^a^) reference category for failed parasitization
