# Supplementary material for: Life‐history traits of the Whiting polyploid line of the parasitoid Nasonia vitripennis
Source: Entomol Exp Appl. 2019 Jul 17;167(7):655–69. doi: 10.1111/eea.12808 (PMC6774307; doi:10.1111/eea.12808)
Supplement: Supplementary file 1 — Table S1. Winners of single‐ and multiple‐mate competitions of Whiting inbred lines for HVRx females. Grayed‐out trials were discarded from analyses because the female of single competition did not mate, or a majority of females in a multiple‐mate competition did not Table S2. Results from the general linearized model for likelihood of females to mate with a haploid or diploid male in the multiple‐mate experiment. The intercept indicates the model incorporating trial number as a random factor. The reference category is set to the haploid male Table S3. Results from the binomial general linearized mixed model for parasitization rate of inbred and outbred Whiting line females. The intercept indicates the model incorporating random factors ‘day’ (host set) and ‘individual’, and the fixed effect ‘ploidy’ or ‘background’. The data for the diploid are relative to the triploid (0a) reference category for failed parasitization [file EEA-167-655-s001.docx]

**Supporting Information**

**Table S1** Winners of single- and multiple-mate competitions of Whiting inbred lines for HVRx females. Grayed-out trials were discarded from analyses because the female of single competition did not mate, or a majority of females in a multiple-mate competition did not mate

| Single competition | |  |  |  |  |
| --- | --- | --- | --- | --- | --- |
| Trial | Male winner |  |  |  |  |
| 1 | Haploid |  |  |  |  |
| 2 | Haploid |  |  |  |  |
| 3 | Haploid |  |  |  |  |
| 4 | Diploid |  |  |  |  |
| 5 | Unmated |  |  |  |  |
| 6 | Unmated |  |  |  |  |
| 7 | Diploid |  |  |  |  |
| 8 | Diploid |  |  |  |  |
| 9 | Haploid |  |  |  |  |
| 10 | Diploid |  |  |  |  |
| 11 | Diploid |  |  |  |  |
| 12 | Unmated |  |  |  |  |
| 13 | Haploid |  |  |  |  |
| 14 | Haploid |  |  |  |  |
| 15 | Diploid |  |  |  |  |
| 16 | Unmated |  |  |  |  |
| 17 | Haploid |  |  |  |  |
| 18 | Haploid |  |  |  |  |
| 19 | Haploid |  |  |  |  |
| 20 | Haploid |  |  |  |  |
| 21 | Haploid |  |  |  |  |
| 22 | Diploid |  |  |  |  |
| Multiple-mate competition | |  |  |  |  |
| Trial | Females mated | Females unmated | Mated with haploid | Mated with diploid | Male winner |
| 1 | 6 | 4 | 0 | 6 | Diploid |
| 2 | 3 | 7 | 3 | 0 | NA |
| 3 | 10 | 0 | 5 | 5 | Tie |
| 4 | 9 | 1 | 8 | 1 | Haploid |
| 5 | 3 | 7 | 3 | 0 | NA |
| 6 | 3 | 7 | 0 | 3 | NA |
| 7 | 7 | 3 | 1 | 6 | Diploid |
| 8 | 8 | 2 | 1 | 7 | Diploid |
| 9 | 9 | 1 | 8 | 1 | Haploid |
| 10 | 6 | 4 | 3 | 3 | Tie |
| 11 | 5 | 5 | 2 | 3 | Diploid |
| 12 | 8 | 2 | 7 | 1 | Haploid |
| 13 | 9 | 1 | 5 | 4 | Haploid |
| 14 | 9 | 1 | 4 | 5 | Diploid |
| 15 | 1 | 9 | 1 | 0 | NA |
| 16 | 6 | 4 | 5 | 1 | Haploid |
| 17 | 8 | 2 | 5 | 3 | Haploid |
| 18 | 1 | 9 | 1 | 0 | NA |
| 19 | 8 | 2 | 2 | 6 | Diploid |
| 20 | 8 | 2 | 4 | 4 | Tie |

**Table S2** Results from the general linearized model for likelihood of females to mate with a haploid or diploid male in the multiple-mate experiment. The intercept indicates the model incorporating trial number as a random factor. The reference category is set to the haploid male

| Experiment (intercept) | Coefficient | Exp (coefficient) | P |
| --- | --- | --- | --- |
| Single mate (BIC = 78302)  Multiple mate (BIC = 515987) | -0.454  0.325 | 0.635  1.384 | 0.38  0.35 |

**Table S3** Results from the binomial general linearized mixed model for parasitization rate of inbred and outbred Whiting line females. The intercept indicates the model incorporating random factors ‘day’ (host set) and ‘individual’, and the fixed effect ‘ploidy’ or ‘background’. The data for the diploid is relative to the triploid (0^a^) reference category for failed parasitization

| Background | | Coefficient | Exp (coefficient) | P |
| --- | --- | --- | --- | --- |
| WPL-inbred (BIC = 13061) | |  |  |  |
|  | Intercept | -1.037 | 0.354 | 0.034 |
|  | Ploidy = 2n | 1.457 | 4.295 | <0.001 |
|  | Ploidy = 3n | 0^a^ |  |  |
| WPL-outbred (BIC = 37017) | |  |  |  |
|  | Intercept | -0.905 | 0.404 | 0.096 |
|  | Ploidy = 2n | 1.950 | 7.032 | <0.001 |
|  | Ploidy = 3n | 0^a^ |  |  |
